# Causes of death in hospitalized children younger than 12 years of age in a Chinese hospital: a 10 year study

**DOI:** 10.1186/s12887-017-0981-y

**Published:** 2018-01-18

**Authors:** Yueniu Zhu, Xiaodong Zhu, Mengyan Deng, Hongxia Wei, Mingjun Zhang

**Affiliations:** Department of Pediatric Critical Care Medicine, Xinhua Hospital affiliated to Shanghai Jiaotong University School of Medicine, Shanghai, 200092 China

**Keywords:** Cause of death, Chronic disease, Congenital abnormality, Hospitalized children, Infectious diseases, Pneumonia

## Abstract

**Background:**

In China, the majority (77%) of urban children die in hospitals. Hospital-based review could provide insight leading to improvements in clinical practice and increase the survival of critically ill children. The aim of the present study is to identify the trends of immediate causes and chronic underlying diseases associated with deaths of children at one of the largest teaching hospitals in China over a period of 10 years (2006–2015).

**Methods:**

A retrospective analysis of data of all children aged 1 month to 11 years who died at Xinhua Hospital between 2006 and 2015. Demographic details, main causes of deaths, and chronic underlying diseases were reviewed.

**Results:**

Case fatality rate was 0.55% (510/93,443) and it represented 0.41–0.80% deaths per year. Overall, the most common immediate causes of deaths in hospitalized children were pneumonia (36.7%), sepsis (13.5%), tumour (11.4%), followed by nontraumatic intracranial or gastrointestinal hemorrhage (10.6%) and cardiac shock (9.6%). Over 70% of the deaths in children were complicated with chronic underlying diseases. Congenital abnormality was the most frequent chronic underlying disease observed in infants (60.3%) and tumour was the main chronic underlying disease in toddlers (31.1%) and older children (44%).

**Conclusions:**

Infectious diseases, especially pneumonia, were the major immediate causes of deaths, and the mortality in the study population decreased with age. Tumour and other noninfectious disease accounted for more deaths in older children. Chronic underlying diseases were found in most deaths of children.

**Electronic supplementary material:**

The online version of this article (10.1186/s12887-017-0981-y) contains supplementary material, which is available to authorized users.

## Background

Mortality rate in children is decreasing worldwide. Mortality in children younger than 5 years has dropped from 11.9 million in 1990 to 7.7 million deaths in 2010 [1]. Official data from the United Nations indicated that China made progress in the significant reduction of neonatal, infant, and childhood deaths during the past few decades [2, 3]. Expecting to achieve the Millennium Development Goal 4, global and local assessments of mortality in newborn and children younger than 5 years are well implemented. However, these estimations do not provide enough information for the identification of the complex causes of deaths in children such as different age groups, immediate cause of death, and chronic underlying diseases contributing to death. The spectrum and characteristics of diseases shift gradually with the growth and development of children. Further investigations on these complex causes of deaths in children could give more information to the healthcare providers to recognize fatal situations. A comprehensive understanding of the causes of death could help improve high quality of hospital care for children and would promote better outcomes.

In China, the majority (77%) of urban children die in hospitals [4]. The number of children hospitalized is increasing, especially in urban centers and large community hospitals. Improving hospital care for seriously ill children is critical for promoting children’s health. Although hospital-based mortality review may not be a whole reflection of deaths from various causes in the general population, it may provide opportunities to examine the immediate and underlying causes contributing to deaths occurring at health facilities. With in-depth investigations, these assessments could forewarn the high-risk patient and renew the sense of commitment to fatal conditions among healthcare staff. The World Health Organization describe hospital-based review as one of the main types of death review that has the potential to provide great improvements in clinical practice and increase the survival of critically ill children [5].

To improve the understanding of the causes of death in children and provide clinical cautionary information to professional healthcare providers, the present study aimed to retrospectively review and identify the trends of immediate causes and chronic underlying diseases associated with deaths of children at one of the largest tertiary-level hospitals in China.

## Methods

### Study design

This retrospective study was carried out to examine the causes of death of paediatric patients who died at the studied hospital. Clinical data of a 10-year period (2006–2015) was collected and examined for children aged 1 month–11 years who died during the study period. This hospital under discussion was the first institution which set up subspecialty departments for children in China. It is the largest general hospital serving as a medical center for local children in Shanghai and also as a tertiary referral centre for children with complex paediatric conditions in the east of China. Each year there are about 10,000 children discharged from the hospital.

The study protocol was approved by the Ethics Committees of the studied hospital, and the need for written informed consent was waived by the committee as all data were used retrospectively and de-identified.

### Data collection and management

Data used in this study were drawn from the hospital information services department databases. These databases prospectively recorded demographic and clinical data on all hospitalized children. The children who died in the emergency department were not included due to incomplete medical records. Demographic details, main causes of deaths, chronic underlying diseases, and other diagnoses during the hospitalization according to the International statistical classification of diseases and related health problems - 10th revision were studied. The main cause of death in the present study referred to the diagnosis of direct cause of death registered on the death certificate, and the chronic underlying diseases referred to the complicated chronic disease diagnosed before death. The medical records of every dead cases were reviewed to confirm the diagnosis on the death certificate. In the study, the direct causes of death were further divided into infectious and noninfectious diseases. The main category of chronic underlying diseases was defined as congenital abnormalities, immunodeficiency and autoimmune diseases, tumours, and others (e.g. malnutrition, obesity, hemophilia, et al.). After reviewing the medical history of the tumour cases, we classify the direct causes of death into tumour while death was caused by the tumour compression or metastasis and complications after aggressive treatment (such as infection or hemorrhage) [Additional file 1]. Clinical data was also divided into three groups by age: 1–12 months old (infants), 1–4 years old (toddlers), and 5–11 years old (older children).

### Statistic analysis

Percentage and proportional mortality ratios were calculated using Microsoft Excel software. Statistical analyses were performed using GraphPad Prism 7.0 for Mac OS X. Data was expressed as mean ± standard deviation (SD). Differences between proportions of groups were analyzed for statistical significance using the chi-square test. The level of statistical significance was set at *p* < 0.05.

## Results

During the study period, 93,443 children were hospitalized; of whom, 510 (0.55%) died. The annual overall fatality rate varied from 0.41% to 0.8%. The majority of deaths in the study occurred in children under 5 years of age (77%) including 42% in infants. Children aged 5–11 years accounted for 23% of the deaths in this study (Table 1).

**Table 1:** Characteristic of children died in Xinhua Hospital during 2006–2015

Characteristic	Value
Age, mean (range)	34mo (1-144mo)
Infants, No. (%)	214 (42)
Toddler, No. (%)	180 (35.3)
Older children, No. (%)	116 (22.7)
Gender, No. (%)	
Male	308 (60.4)
Female	202 (39.6)
Immediate cause of death, No. (%)	
Infectious diseases	311 (60.9)
Pneumonia	187 (36.7)
Sepsis	69 (13.5)
CNS infection	39 (7.6)
Diarrhoea	16 (3.1)
Non infectious diseases	192 (37.7)
Non traumatic intracranial/gastrointestinal hemorrhage	54 (10.6)
Cardiac shock	49 (9.6)
Tumour	58 (11.4)
Accident	31 (6.1)
Other	7 (1.4)
With chronic underlying diseases, No. (%)	391 (76.7)
Congenital abnormalities	205 (40.2)
Immunodeficiency and Autoimmune diseases	54 (10.6)
Tumour	125 (24.5)

Overall, the most common causes of deaths in the hospitalized children were pneumonia (36.7%), sepsis (13.5%) and tumour (11.4%), and followed by non-traumatic intracranial or gastrointestinal hemorrhage (10.6%), cardiac shock (9.6%), central nerve system infection (7.6%), accident (6.1%), and diarrhoea (3.1%, Table 1).

### Distribution of immediate cause of death

The main causes of deaths based on the ages are shown in Table 2. Infectious diseases caused the majority of the deaths in children under 5 year age. The overall deaths caused by infectious diseases decreased significantly with age, from 72.4% in infants to 44.8% in elder children (*p* < 0.0001). Of all the infectious diseases, pneumonia, sepsis, and CNS infection were the main causes of death throughout childhood. The proportion of deaths from pneumonia also decreased with this reduction, from 51.4% in infants to 20.7% in older children (*p* < 0.0001). On the contrary, the overall death due to noninfectious disease increased significantly with age. Of all these noninfectious diseases, non-traumatic intracranial or gastrointestinal hemorrhage, cardiac shock, tumour, and accident were the main causes of death. The proportion of deaths from tumour and accidents increased dramatically with age.

**Table 2:** Cause of death distribution by age in children at Xinhua Hospital during 2006–2015

	Infants (*n*/214)		Toddlers (*n*/180)		Older children (*n*/116)		*p* value^a^
	n	%	n	%	n	%	
Infectious diseases	155	72.4	104	57.7	52	44.8	<0.0001
Pneumonia	110	51.4	53	29.4	24	20.7	<0.0001
Sepsis	25	11.7	29	16.1	15	12.9	0.4306
CNS infection	11	5.1	17	9.4	11	9.5	0.1939
Diarrhoea	9	4.2	5	2.8	2	1.7	0.4399
Non infectious diseases	56	26.2	73	40.6	63	54.3	<0.0001
Non traumatic intracranial/ gastrointestinal hemorrhage	17	8.0	21	11.7	16	13.8	0.2165
Cardiac shock	24	11.2	14	7.8	11	9.5	0.5136
Tumour	8	3.7	24	13.3	26	22.4	<0.0001
Accident	7	3.3	14	7.8	10	8.6	0.0751
Other	3	1.4	3	1.7	1	0.9	0.8438

^a^Chi-squared test

In infants, pneumonia caused the largest proportion of deaths (51.4%), while sepsis and cardiac shock were the second and third main causes. In toddlers pneumonia was also the leading cause of death, while sepsis and tumours contributed substantially. In older children, despite the proportion of pneumonia deaths decreasing to 20.7%, it remained a main cause of death, followed by tumour, non-traumatic intracranial or gastrointestinal hemorrhage, and sepsis (Table 2).

### Underlying chronic disease related to death

**Fig. 1:**
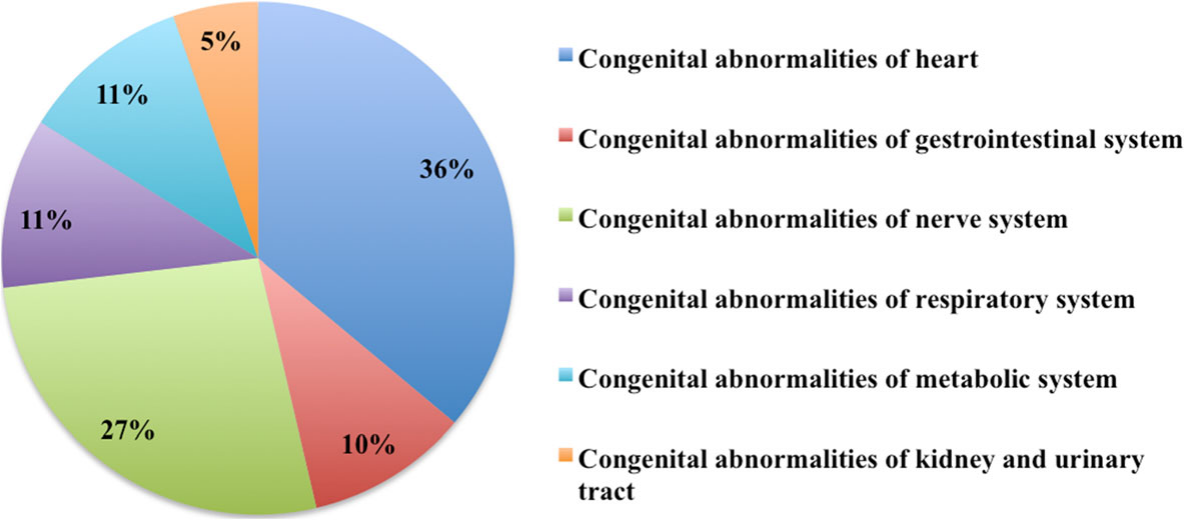
Distribution of congenital diseases in children who died at Xinhua Hospital during 2006–2015

Between 74.1 and 79.9% of the deaths in children were complicated with chronic underlying diseases (Table 3). A congenital abnormality was the most frequent chronic underlying disease observed in infants, and this proportion decreased dramatically with age. Of the deaths in infants, 60.3% were complicated with congenital abnormalities, while the proportion decreased to 31.1% in toddlers and 17.2% in older children (*p* < 0.0001). Congenital abnormalities of the heart and the nervous system were the main abnormalities found in the deaths of children (Fig. 1). The main direct causes of death in the children with congenital abnormalities were infectious diseases (*n* = 153, 74.6%), while the other children with congenital abnormalities died from organ dysfunction due to the abnormalities.

**Table 3:** Distribution of chronic underlying diseases of children who died in Xinhua Hospital during 2006–2015

	Infants (*n*/214)		Toddler (*n*/180)		Older children (*n*/116)		*p* value^a^
	n	%	n	%	n	%	
With chronic underlying diseases	171	79.9	134	74.4	86	74.1	0.3384
Congenital abnormalities	129	60.3	56	31.1	20	17.2	<0.0001
Immunodeficiency and Autoimmune diseases	18	8.4	22	12.2	14	12.0	0.3970
Tumour	18	8.4	56	31.1	51	44.0	<0.0001
Other	6	2.8	0	0	1	0.9	0.0506
Without chronic underlying diseases	43	20.1	46	25.6	30	25.9	0.3384

^a^Chi-squared test

On the other hand, a tumour was the main chronic underlying disease in the children that died at more than 1 year of age and the proportion increased with age. Only 8.4% of the deaths in the infant group were complicated with a tumour, while the proportion increased significantly to 31.1% in toddlers and 44% in older children (*p* < 0.0001). The main direct cause of death in children with a tumour was therapy related-complications (*n* = 68, 54.4%), while the rest died from organ dysfunction due to tumour compression or metastasis. The therapy related-complications included serious infection (*n* = 45, 36%) and hemorrhage (*n* = 23, 18.4%).

## Discussion

The current study aimed to identify the main causes and complications underlying diseases associated with deaths in children. Children hospitalized at a tertiary hospital represent the important subgroups to have high risk of mortality. Reviewing hospital deaths in a structured way could provide good understanding of causes of deaths and allow improving of better interventions.

In the present study, 77% of deaths occurred in children under the age of five including 42% in children under 1 year of age. This is similar to the studies that mentioned around 80% of deaths occurred in children who were under 5 years in hospitals of developing countries [6, 7], and infant mortality accounting for 30–50% of all deaths among children younger than 5 years of age in Asia [8, 9] These data suggest the vulnerability of children in these ages. The number of dead case in boys is a bit more than that in girls. This might partially due to the Chinese tradition of preference boys to girls while more seriously ill boys were transferred to the senior medical center.

### Cause of death in hospitalized children

The study results showed that infectious diseases, accounting for 61% of total death, were the leading causes of death in children under 5 years age. The infectious disease category in the study included pneumonia, sepsis, CNS infection, and diarrhoea. The proportion of infectious diseases and pneumonia decreased significantly with age. These trends were similar to that described globally and regionally [10, 11]. These shifts may associate with gradual maturity of children’s immune system and also benefit from increased immunization procedures introduced to older children (A serial of vaccinations are compulsory or recommended to children from birth to 8 years. The compulsory vaccines include Bacillus Calmette-Guerin, Hepatitis B, poliomyelitis, diphtheria/pertussis/Tetanus, measles, Japanese encephalitis, epidemic encephalitis, et al.).

Pneumonia is the leading infectious causes of childhood morbidity and mortality worldwide [1, 12]. With an estimated 146–159 million new episodes per year in developing countries, pneumonia is estimated to cause approximately 2 million deaths among children globally [13]. In China, pneumonia is also the most frequent cause of deaths in children [14]. In the present study, pneumonia was, as expected, the ‘top killer’ of children. The study suggested that younger children and children with chronic underlying diseases (particularly congenital diseases) were more likely to die from pneumonia. It has been proved that different organisms could be isolated from these susceptible children [15]. In infants, pneumonia mostly results from respiratory viruses and bacterial etiologies such as *Streptococcus pneumonia*. Children with chronic disease often acquire pneumonia caused by *Klebsiella*, *Enterobacter*, *Pseudomonas*, and *Acinetobacter* [15]. Results of a global study suggested that several preventive and therapeutic strategies such as appropriate antibiotics, vaccination, and preventive zinc supplementation were effective in the control of pneumonia [16]. This simulation also estimated that 51% of pneumonia deaths would be saved by 2025 if targeted strategies are implemented at present.

Although diarrhoea was described as a leading cause of death in children globally [1, 10], diarrhoea was not a common cause of child deaths in all age groups (only 1.7–4.2%) in this study. The distribution is consistent with the national mortality report [11, 14]. The lower Chinese mortality contrasted to the global results might relate the common cultural practice of boiling water, introduction of rotavirus vaccine, and other hygiene practices in China.

### Chronic underlying disease related to death

The present study found that more than 70% of the deaths in children were complicated with chronic underlying diseases. The number of morbidity of chronic disease in the study is far more prevalent than that in the general population which is varied from 1.5–5% [11, 17]. This suggested that children with complex chronic conditions were a small subset of children that accounted for more than half of childhood death from medical causes. It was estimated that around 70% of no injury deaths among children ages 1–4 and and more than 80% of deaths among all school-age children were result of chronic causes [18]. These data were similar with the present study results, and this suggests that chronic underlying disease may play an important role in the death of children.

This study found that congenital abnormality was the most frequent chronic underlying disease observed in children under 1 year of age, and the proportion decreased dramatically with increase in age. Tumours became the leading chronic underlying disease in children aged 5–11 years, followed by congenital abnormalities and immune diseases. Even with advances in corrective surgery, congenital heart disease remains the leading cause of death in children with congenital malformations [15]. In the present study, congenital heart disease attributed 36.1% in all congenital abnormalities. Acute infectious disease is an important cause of death in children with chronic illness in the developed countries [19]. In the study, about three quarters of the congenital abnormality children died from infectious disease, and rest of the population died from organ decompensate due to congenital abnormality. The trends indicated that children complicated with congenital abnormalities are on the risk of life-threatening infection and require specialized care to prevent early deaths. The hospital medical staff needs to be alerted to the signs or symptoms which might be relevant to infection in these children.

Tumours remain a major cause of death in children [11, 20], although outcomes have considerably improved over the past few decades (five-year survival increasing from approximately 40% in the 1970s to approximately 80% in the 2000s [21, 22]). The morbidity and mortality of a tumour also increases with age [11]. In the present study, up to 44% of the deaths of children in 5–11 years group were related to a tumour. As the development of oncotherapy and do-not-attempt-resuscitation orders remain uncommon in paediatric practice, more children with tumours die in the hospital [23, 24]. It was reported that the main causes of death in hospitalized children with a tumour were severe sepsis, pneumonia, and respiratory failure [25, 26]. The present study indicated that at least 54.4% of deaths in these children were due to complications from oncotherapy. These complications included serious infection (36%) and hemorrhage (18.4%). Several strategies such as admission to palliative intensive care unit, providing organ support and early aggressive hemodynamic assessment were proved to be effective in patients at risk of these life-threatening complications [27, 28]. Careful observation and early intervention of these patients could increase the survival and make them benefit more from the advances in oncotherapy.

The relationship between chronic underlying diseases and deaths has, to our knowledge, not been mentioned much in previous death reviews. The present study indicated that most children died with chronic underlying diseases. Congenital abnormalities were the major chronic underlying disease in infants and a tumour was the main chronic disease in children more than 1 year of age. Infection diseases and therapy related-complications were often life-threatening in these children. Thus, this study may help to highlight the improvement of clinical evaluation and management in these children with complex clinical condition.

### Limitation

The major limitation of this study was its retrospective design. Although some parts of the results were consistent with the global and national data of mortality, these results should be interpreted bearing in mind that they only included deaths occurring at a tertiary health care facility. This study discussed the main diseases related to the death of children. Further investigation should be carried out to explore the clinical presentation and therapy strategies related to death in hospitalized children. With this information, healthcare providers could recognize fatal situation more efficiently and treat these patients more effectively.

## Conclusion

Infectious diseases, especially pneumonia and sepsis, were the major immediate causes of death, and the mortality decreased with age in children more than 1 year of age. In contrast, a tumour and accident accounted for more deaths in children more than one-year of age. Chronic underlying diseases were found in most deaths of children. Congenital abnormalities were the major chronic underlying disease in infants and a tumour was the main chronic disease in children more than 1 year of age. Infection diseases and therapy-related complications were often life-threatening in these children.

## Additional file

Additional file 1: Disease category. (DOC 36 kb)
