# Supplementary material for: Causes of death in hospitalized children younger than 12 years of age in a Chinese hospital: a 10 year study
Source: BMC Pediatr. 2018 Jan 18;18:8. doi: 10.1186/s12887-017-0981-y (PMC5773040; doi:10.1186/s12887-017-0981-y)
Supplement: Additional file 1: — Disease category. (DOC 36 kb) [file 12887_2017_981_MOESM1_ESM.doc]

Disease category

| Category | Diseases refered |
| --- | --- |
| Immediate cause of death |  |
| Infectious diseases |  |
| Pneumonia | Pneumonia (with different pathogen) |
| Sepsis | Sepsis, Septicemia |
| CNS infection | viral encephalitis, bacterial meningitis, tubercular meningitis |
| Diarrhoea | Diarrhea, acute gastroenteritis |
| Non infectious diseases |  |
| Non traumatic intracranial/  gastrointestinal hemorrhage | upper gastrointestinal hemorrhage, gastrointestinal hemorrhage, non traumatic intracranial hemorrhage, hemorrhage shock |
| Cardiac shock | Cardiac shock (appeared as direct cause of death on death certificate)+ cardiac disease mentioned in the following part |
| Tumour | Leukemia, lymphoma and other solid tumors appear on death certificate, review the medical record to judge whether death directly caused by tumour compression or metastasis |
| Accident | traffic accident, falling off building, drowning, poisoning |
| With chronic underlying diseases, No. (%) |  |
| Congenital abnormalities | congenital abnormalities include cardiovascular, respiratory, urinary, nerve, gastrointestinal, metabolic system |
| Immunodeficiency and  Autoimmune diseases | Immunodeficient disease, autoimmune disease (such as SID, SLE, Hashimoto’s thyroiditis, dermatomyositis, autoimmune encephalitis) |
| Tumour | Leukemia, lymphoma, other solid tumour appear on the death certificate, review the medical record to judge whether death directly caused by aggressive therapy |
| Other | Malnutrition, obesity, hemophilia |
